# Experience-stratified efficacy of 3D printed models in assisting orthopedic residents with pelvic ring injury classification: a comparative analysis

**DOI:** 10.1186/s13018-025-06479-4

**Published:** 2025-12-16

**Authors:** Chaoqun Wang, Ulrich Stöckle, Shengnan Dong, Chengfei Meng, Feifei Tong, Zexi Ling, David Alexander Back

**Affiliations:** 1 Department of Traumatic Orthopaedics, The Affiliated Hospital of Wuhan Sports University, NO.279 On Luoyu Road, Hongshan District, Wuhan City, 430079 Hubei Province China; 2 Center for Musculoskeletal Surgery, Charité – Universitätsmedizin Berlin, Corporate member of Freie Universität Berlin and Humboldt-Universität Zu Berlin, Augustenburgerplatz 1, 13353 Berlin, Germany

**Keywords:** Pelvic fractures, Three-dimensional, Classification, Orthopedic residents, Education tool

## Abstract

**Background:**

Despite the utility of three-dimensional (3D) printing in teaching complex anatomy, its application in pelvic ring injury education remains limited. This study aimed to determine whether 3D printed models improve the accuracy of pelvic fracture AO/OTA classification over conventional imaging, and to assess how this effect differs between junior and senior orthopedic residents.

**Methods:**

Imaging data of 10 pelvic ring injury cases were collected to produce corresponding 3D models. Four rounds of classification tests were conducted among orthopedic residents in our department using different imaging modalities: two-dimensional (2D) images alone, 2D images + 3D computed tomography (CT) reconstructions, 2D images + 3D reconstructions + 3D printed models, and 3D printed models alone. The classification accuracy reaching subgroup level and time consumption were analyzed to assess the utility of 3D models. Additionally, a subjective questionnaire was used to gather participants’ perceptions of 3D models as educational and diagnostic tools.

**Results:**

Among 45 enrolled participants, 18 who completed all rounds were included and grouped by experience: junior (≤ 5 years, *n* = 10) and senior (> 5 years, *n* = 8). Compared to using conventional digital imaging, both adjunctive and independent use of 3D models improved classification accuracy in junior group (adjunctive: median difference = 25.00%, 95% CI: 10.00 to 45.00%, Bonferroni-adjusted *P* = 0.034; independent: median difference = 25.00%, 95% CI: 10.00 to 40.00%, Bonferroni-adjusted *P* = 0.034). Among senior residents, independent use of 3D models did not improve classification accuracy (median difference = 10.00%, 95% CI: − 10.00 to 15.00%, Bonferroni-adjusted *P* = 1.000), and adjunctive use significantly reduced accuracy (median difference = 30.00%, 95% CI: 10.00 to 55.00%, Bonferroni-adjusted *P* = 0.016). Moreover, Both modes of 3D model application significantly increased time consumption relative to conventional imaging (adjunctive: median difference =  − 585.50 s, 95% CI: − 1150.01 to − 283.50, Bonferroni-adjusted *P* < 0.001; independent: median difference =  − 211.00 s, 95% CI: − 344.50 to − 82.00, Bonferroni-adjusted *P* = 0.039). Subjective feedback revealed overall positive attitude toward 3D models, with a stronger preference for their role in educational contexts over clinical decision-making.

**Conclusion:**

3D printed models exhibit experience-stratified difference in pelvic fracture classification, improving accuracy for junior residents while reducing it for seniors. Both adjunctive and independent use of 3D models increased time consumption compared to digital imaging alone. Participants predominantly viewed 3D models as classification supplements rather than clinical decision tools, with no consensus on their surgical utility.

## Introduction

The pelvic ring is a circular structure composed of the sacrum, coccyx, two innominate bones, and ligaments connecting them. In traumatic orthopedics context, the pelvic ring is usually divided into two parts by a virtual line traversing the bilateral acetabulum [1]. The anterior ring extends from the anterior acetabular column along the pubic ramus to the pubic symphysis, whereas the posterior ring is mainly defined as the continuous and micro-mobile structure consisting of the the bilateral sacroiliac joints and sacrum [2]. Since the posterior pelvic ring plays a major role in maintaining the stability of the pelvic ring [1], isolated injuries to the anterior pelvic ring are considered stable, whereas complete or incomplete injuries to the posterior pelvic ring reveal a significant disruption of the stability of the pelvic ring, and are often combined with other injuries including damage to nerves, blood vessels, and visceral organs [3–7].

The evaluation of the stability of the pelvic ring is closely associated with the choice of subsequent therapy. Currently, conservative treatment is advocated for stable pelvic ring injuries, early pelvic stabilization surgery is advocated for completely unstable pelvic ring injuries, and cases in between need to be considered in conjunction with other factors in order to determine whether or not to recommend surgical treatment [2]. Moreover, accurate diagnosis and classification of pelvic ring injuries is a prerequisite for the determination of an optimal therapy. On the one hand, although not all pelvic ring injuries require surgical treatment, the key issue is to make sure that our diagnosis and classification are accurate before determining the conservative treatment, which means that we must make sure that we do not miss any unstable pelvic ring injuries that require surgical management. On the other hand, for unstable pelvic ring injuries requiring surgery, accurate preoperative diagnosis and classification will help us to analyze the injury mechanism and develop an optimal surgical plan, particularly in selecting the most appropriate surgical approach. As highlighted by the recent network meta-analysis by Ramadanov et al. [8], pararectus and intrapelvic approaches for acetabular fractures are superior to the ilioinguinal approach in reducing overall complications and intraoperative blood loss, and achieving better reduction quality. This evidence directly confirms that precise preoperative assessment directly contributes to reducing intraoperative risks and improving postoperative results.

Currently, the most commonly used screening tests for diagnosing pelvic ring injuries are X-rays and computed tomography (CT). Taking a pelvic plain radiograph is a quick and convenient way to determine whether the pelvic ring is significantly deformed or unstable. Pelvic plain radiographs are recommended as a rapid diagnostic tool for early intervention especially when treating hemodynamically unstable patients [1–3, 7]. However, due to the irregular and overlapping morphology of the bony pelvic structures and the interference of abdominal contents in the photographs, it is usually difficult to acquire a well defined view of the pelvic ring, which makes it challenging to formulate an accurate diagnosis and classification on the sole basis of plain radiographs. Furthermore, the accuracy of plain radiographs is extremely poor especially when assessing posterior pelvic ring injuries [9]. A CT scan helps to improve diagnostic and classification accuracy by clearly visualizing the fracture line and displacement in any desired section. Therefore, CT scans are essential for assessing the stability of the pelvic ring and are regarded as the gold standard for diagnosing pelvic ring injuries [2, 4, 7]. However, due to the lack of three-dimensional (3D) anatomical knowledge of pelvis, minimally trained orthopedic residents often struggle to accurately conceptualize the actual 3D geometry of pelvic ring injuries from two-dimensional (2D) images. Consequently, accurate diagnosis and classification remain a challenge to them. Additionally, they may even be misled by the clearly defined fracture lines on certain special tomographic sections and misidentify an incomplete posterior pelvic ring injury as a complete injury.

With the rapid development of technology, 3D printing technology has now been widely used in clinical practice [10–14], and its application in orthopedic context has also achieved considerable results in recent years [15–20]. A typical example is the use of 3D printing technology for preoperative planning of orthopedic surgeries, such as the manufacture of osteotomy templates, fracture reduction guides, and individually customized internal fixation implants or articular prostheses [16, 21–26]. Moreover, 3D printing technology has also been used as an education tool for complex anatomical structures, and many studies have reported significant effectiveness [27–33]. However, to the best of our knowledge, despite the well-established educational benefits of 3D printed models for acetabular fractures [34, 35], research on the use of 3D printed models as a diagnostic and educational tool for pelvic ring injuries remains limited. While Yan et al. [36] recently demonstrated the educational value of 3D printed models for teaching Young-Burgess classification to fourth-year medical students of a 5-year clinical medicine program, this study extends this investigation by evaluating the efficacy of 3D models in facilitating AO/OTA classification [37] specifically among orthopedic residents, who have substantially more baseline diagnostic experience than medical students. We believe that such a study design is more compatible with the clinical reality and more referential for assessing the clinical educational and diagnostic effectiveness of 3D printed models. Our primary objective was to evaluate whether 3D printed models could improve accuracy of pelvic injury AO/OTA classification by orthopedic residents compared to conventional imaging alone, and whether such benefits differed between junior and senior residents. The secondary objectives included investigating whether 3D models helped reduce the time required for classification decisions and collecting participants’ perceptual feedback on the effectiveness of 3D models in assisting the classification of pelvic ring injuries.

## Methods

### Clinical data extraction and generation of 3D printed models

Ten representative cases of pelvic injuries were selected and anonymized. The AO/OTA classification and injury mechanism were verified by 2 senior orthopedic professors specializing in pelvic and acetabular fracture surgery. Each surgeon independently classified all cases based on the complete set of imaging data and 3D printed models. In the event of a disagreement on group and subgroup, a consensus was reached through a dedicated discussion session. The adjudicated classification, which was defined down to the AO/OTA subgroup level, served as the definitive reference for determining the correctness of the residents’ answers.

The distribution of AO/OTA classification among the selected cases was as follows: Type A in 1 case (subgroup A2.1), Type B in 2 cases (both subgroup B2.1), and Type C in 7 cases (including 2 cases of C1.2, 2 cases of C3.3, with one case each of C1.3, C2.3, and C3.2).

The anteroposterior pelvic X-rays and 2D CT scans (axial, sagittal, and coronal views) were obtained from our digital imaging database. Additionally, CT data of each case was exported to a DICOM (digital imaging and communications in medicine) file, which was then imported into the Materialise Mimics Medical (Version 23.0, Materialise NV, Belgium) to reconstruct 3D images (anteroposterior, inlet, outlet, iliac oblique, obturator oblique, and 2 other characteristic views), followed by processing to Stereolithography (STL) file. These STL files were collected and transferred to the 3D printer (EOS Formiga P110 Velocis, EOS GmbH, Germany). The pelvic fracture models were then fabricated using selective laser sintering (SLS) technology with PA 2200 polyamide powder (EOS GmbH, Germany). The layer thickness was set at 0.1 mm. Following printing, the models underwent standard post-processing procedures, including depowdering and bead blasting, to remove residual powder and achieve a smooth surface finish suitable for handling and anatomical assessment. It should be noted that due to variations in pelvic size among patients, all models were uniformly scaled down to 75% of their original dimensions during printing to ensure compatibility with the 3D printer’s maximum build volume while maintaining the clarity of key anatomical structures.

### Study design

The study was structured into four rounds based on different types of imaging data provided, and participants were expected to give their answers about the classification of each case according to these data.

In the first round, participants were provided with X-rays (anteroposterior view) and 2D CT images (representative axial, sagittal, and coronal views) for each case. The second round extended the first by incorporating additional 3D images (representative anteroposterior, inlet, outlet, iliac oblique, obturator oblique, and two other characteristic views). In the third round, participants were provided with 3D printed models in addition to the imaging data available in the second round. The fourth round differed substantially in that only 3D printed models were available. Table 1 presents the data characteristics and combination strategies for each testing round, using a 61B2.1 case as a representative example. We generated a unique QR (quick response) code for each testing round using the SurveyPluto platform. Participants scanned the QR code with their smartphones to access the response interface, at which point the system automatically initiated timing. Upon completing the classification of all cases and clicking the submit button, the timer stopped automatically. This platform enabled the efficient collection of both time consumption data and classification responses.

**Table 1:**
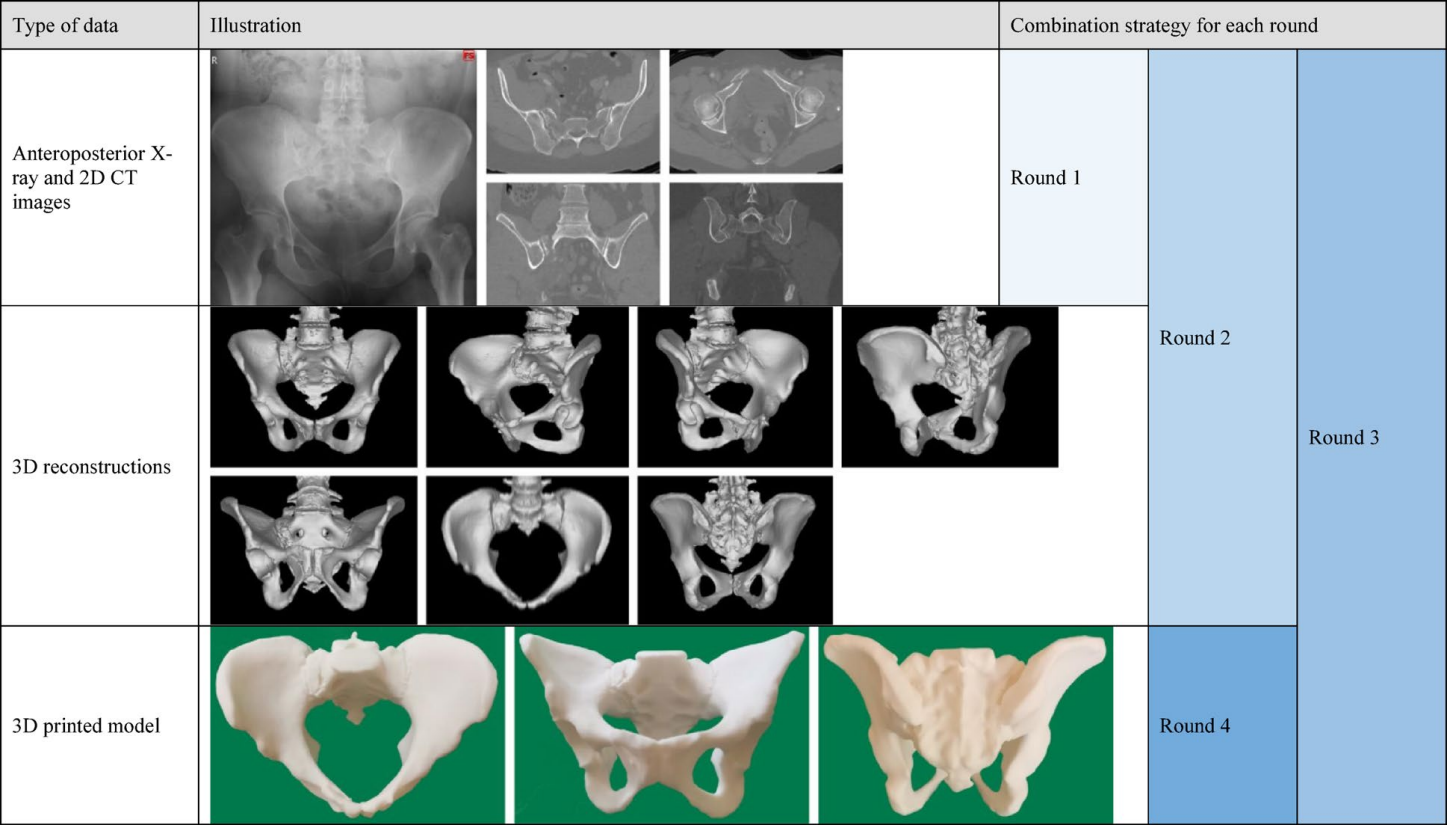
Demonstration of data characteristics and combination strategy for each round using a 61B2.1 case as example

Following the completion of these four testing rounds, participants were invited to complete a subjective questionnaire consisting of eight questions to capture their perceptual feedback on the effectiveness of 3D printed models in assisting pelvic ring injuries classification.

### Participants and data collection

All orthopedic residents from the Musculoskeletal Surgery Center at the Virchow Campus of Charité Hospital, a Level I trauma center, were eligible to participate voluntarily in this study after providing written informed consent. Participants who completed all testing rounds and the subjective feedback questionnaire were included as eligible subjects in the final analysis. Data on participants’ clinical experience, time consumption, classification answers, and subjective feedback were systematically collected. During data processing, eligible participants were ultimately stratified into two groups based on their years of working experience: a senior group (> 5 years) and a junior group (≤ 5 years). The responses with the correct type, group, and subgroup were considered accurate and used for the classification accuracy calculation. A participant’s accuracy rate was calculated as the percentage of his correct responses per round. Since each round consisted of 10 cases, this was equivalent to the number of correct answers multiplied by 10.

### Statistical analysis

Statistical analysis was performed using IBM SPSS Statistics version 27.0 (IBM Corp., released 2020, Armonk, NY, USA). Normality and homogeneity of variance were assessed using the Shapiro–Wilk test and Levene’s test, respectively. Continuous data conforming to a normal distribution are presented as mean ± standard deviation, while skewed data are reported as median with interquartile range (IQR). Categorical variables are summarized as counts and percentages.

All analyses were planned a priori based on the study objectives. Given the repeated-measures design and the need for multiple pairwise comparisons, our statistical tests were organized into distinct families to control the family-wise error rate effectively. The analysis plan was structured as follows:

- *Family 1*: Overall differences in classification accuracy across the four rounds were examined using one-way repeated-measures ANOVA. Pairwise post-hoc comparisons were conducted with Bonferroni correction to maintain the family-wise error rate.
- *Family 2*: To examine whether performance trends across rounds differed by experience level, accuracy data were analyzed separately for senior and junior groups. As the data violated assumptions of normality or homogeneity of variances, the Friedman test (nonparametric repeated-measures ANOVA) was applied for each group, with Bonferroni adjustment used in post-hoc analyses.
- *Family 3*: Differences in classification accuracy between senior and junior groups within each round were assessed. For Rounds 1, 2, and 4, the independent-samples t-test was used. For Round 3, where the junior group’s accuracy data deviated from normality, the Mann-Whitney U test was applied instead.
- *Family 4*: Differences in time consumption across the four rounds were analyzed irrespective of experience level. Due to the skewed distribution of time data, the Friedman test was employed, followed by Bonferroni-corrected pairwise comparisons.
- *Family 5*: Interobserver agreement between the two senior surgeons who established the classification reference standard was evaluated using Cohen’s kappa (κ). Interpretation followed the guidelines proposed by Landis and Koch [38]: κ value of < 0 (poor reliability), 0.00 to 0.20 (slight), 0.21 to 0.40 (fair), 0.41 to 0.60 (moderate), 0.61 to 0.80 (substantial), and 0.81 to 1.00 (almost perfect). To provide a comprehensive assessment of concordance, agreements were assessed across all levels of the AO/OTA classification system, including type, group, and subgroup.

Mean differences (MD) and median differences (MedDiff) with their corresponding 95% confidence intervals (CI) were reported where appropriate, and a *P*-value < 0.05 was considered statistically significant.

## Results

### Interobserver reliability of the classification reference

The interobserver reliability for the AO/OTA classification between the two expert surgeons was excellent based on X-ray and CT imaging. Cohen’s kappa statistics indicated perfect agreement at the type level (κ = 1.00), excellent agreement at the group level (κ = 0.89), and substantial agreement at the subgroup level (κ = 0.68), with all *P*-values < 0.001. When 3D models were used, perfect agreement (κ = 1.00) was achieved at type and group levels (both *P* < 0.001), and excellent agreement was observed at subgroup level (κ = 0.89, *P* < 0.001). These results not only affirm the reliability of the reference standard but also confirms the sufficient diagnostic fidelity of the 75%-scale 3D models, thereby supporting their use in this classification test study.

### Participant selection and grouping

**Fig. 1:**
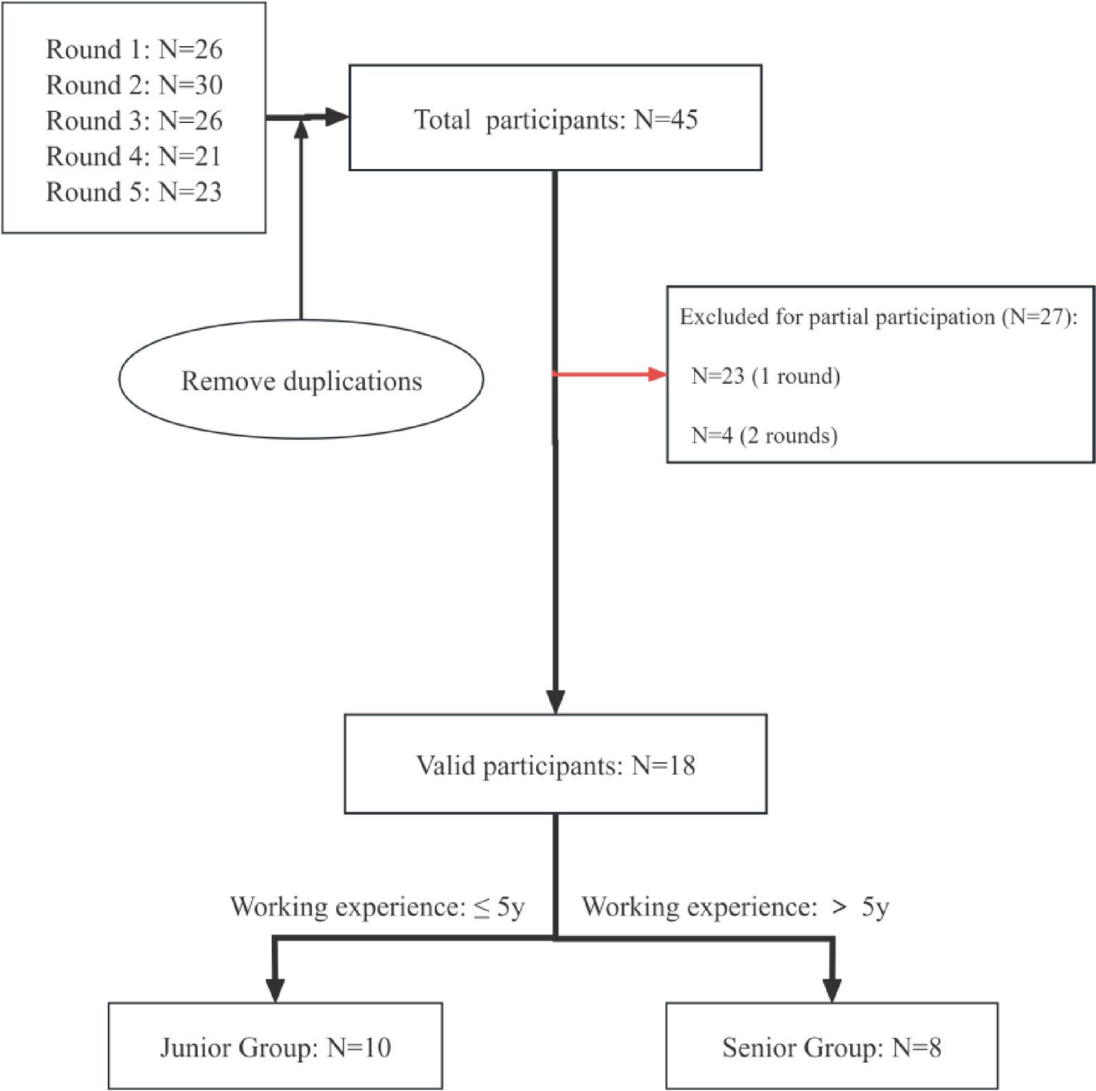
Participant screening flowchart

A total of 126 test responses from 45 individuals were initially collected for this study, including data from four rounds of testing and one subjective feedback questionnaire. After excluding participants who did not complete all five study components, 18 valid participants were included in the final analysis. These participants were further stratified into two groups according to their years of working experience: a junior group (≤ 5 years, *n* = 10) and a senior group (> 5 years, *n* = 8). The detailed selection process is illustrated in Fig. 1.

### Accuracy analysis and comparisons

Comparative analyses of classification accuracy were conducted across all four testing rounds, with subgroup analyses stratified by clinical experience and intra-round comparisons between the two experience groups.

#### Inter-round accuracy comparisons

**Fig. 2:**
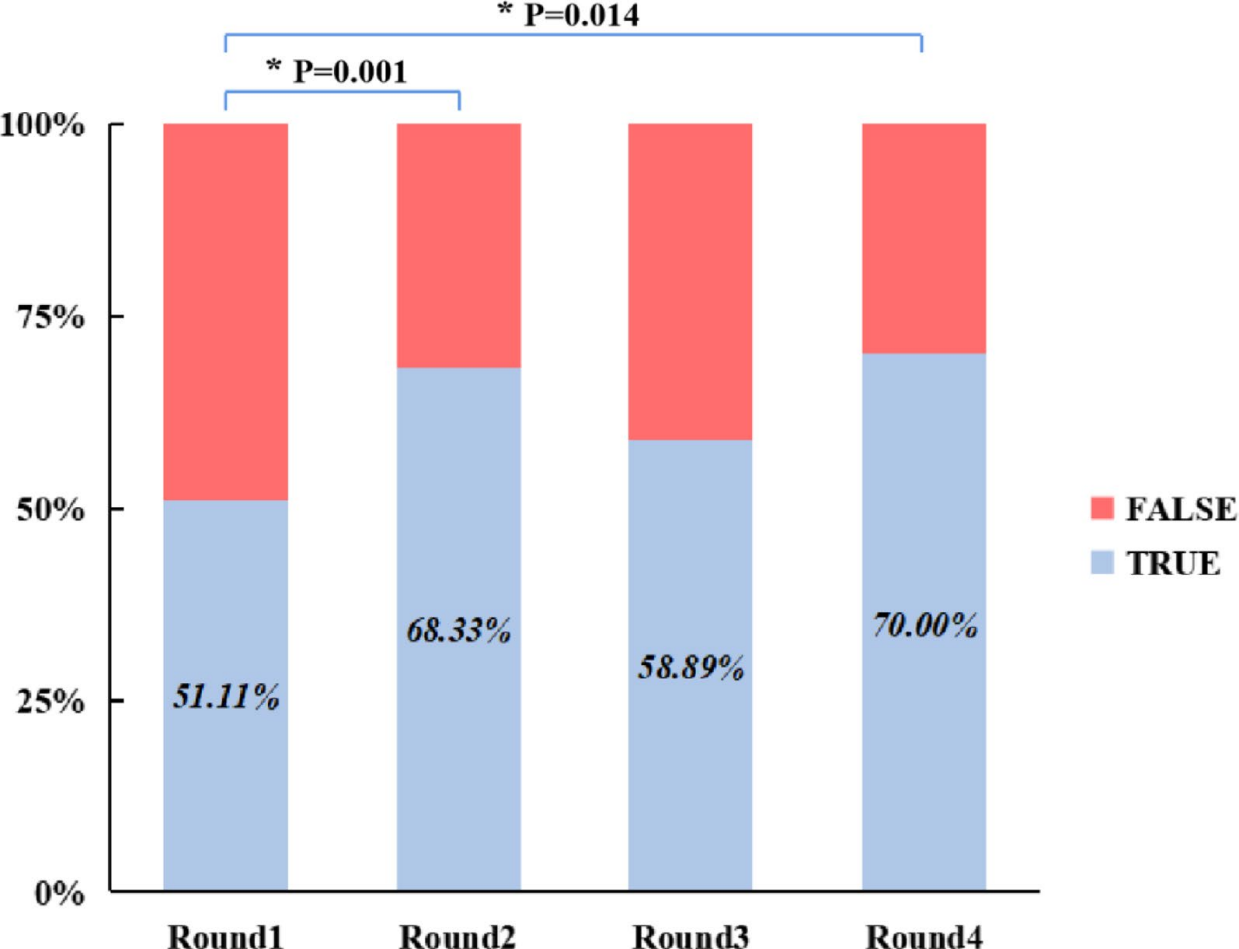
Overall classification accuracy across the four test rounds. Statistically significant differences identified through post-hoc pairwise comparisons are indicated with brackets. Groups without brackets did not differ significantly. An asterisk (*) denotes that the *P*-value has been adjusted using the Bonferroni correction

The overall accuracy rates for Rounds 1, 2, 3, and 4 were 51.11% ± 26.54%, 68.33% ± 18.87%, 58.89% ± 21.39% and 70.00% ± 15.34%, respectively. A one-way repeated-measures ANOVA was conducted to compare the overall accuracy across the four testing rounds. Mauchly’s test of sphericity indicated a violation of the sphericity assumption (W = 0.182, χ^2^ = 26.799, *P* < 0.001); therefore, the Greenhouse–Geisser correction was applied (ε = 0.519). The analysis revealed a significant main effect of testing round (F = 4.099, *P* = 0.037, ηp^2^ = 0.194, indicating that accuracy varied significantly across testing rounds. Post-hoc pairwise comparisons with Bonferroni adjustment further revealed that the overall accuracy in Round 1 was significantly lower than that in Round 2 (MD =  − 17.22%, 95% CI: − 28.23 to − 6.22%, adjusted *P* = 0.001) and Round 4 (MD =  − 18.89%, 95% CI: − 34.69 to − 3.09%, adjusted *P* = 0.014). In contrast, the difference between Rounds 1 and 3 was not statistically significant (MD =  − 7.78%, 95% CI: − 33.60 to 18.04%, adjusted *P* = 1.000). Although accuracy in Round 4 was numerically higher than in Round 2 (MD = 1.67%, 95% CI: − 9.19 to 12.52%, adjusted *P* = 1.000) and Round 3 (MD = 11.11%, 95% CI: − 6.10 to 28.30%, adjusted *P* = 0.425), neither difference reached statistical significance. Additionally, while Round 2 showed approximately 10% higher accuracy than Round 3, this difference was also not statistically significant (MD = 9.44%, 95% CI: − 14.37 to 33.26%, adjusted *P* = 1.000). Details are shown in Fig. 2.

**Fig. 3:**
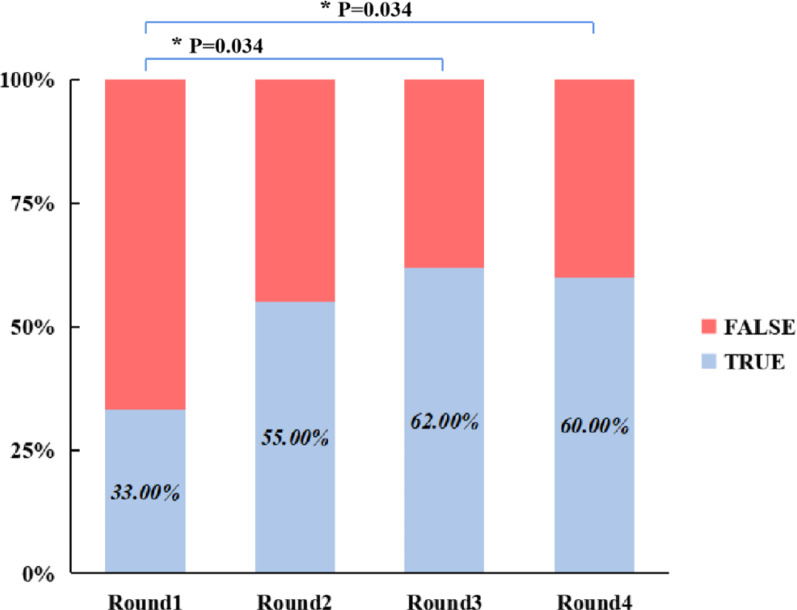
Comparison of classification accuracy across test rounds within the junior group. Statistically significant differences identified through post-hoc pairwise comparisons are indicated with brackets. Groups without brackets did not differ significantly. An asterisk (*) denotes that the *P*-value has been adjusted using the Bonferroni correction

In the junior group, accuracy rates across the four rounds were 33.00% ± 20.58% (median = 35.00%, IQR: 22.50–45.00%), 55.00% ± 11.79% (median = 55.00%, IQR: 50.00–62.50%), 62.00% ± 19.32% (median = 60.00%, IQR: 47.50–67.50%), and 60.00% ± 10.54% (median = 60.00%, IQR: 50.00–70.00%). Since the accuracy in Round 3 violated the normality assumption (*P* = 0.040), the Friedman test was used to compare the four rounds, with Bonferroni correction applied to post-hoc pairwise comparisons. The results indicated a statistically significant effect of testing rounds on accuracy (χ^2^ = 13.098, *P* = 0.004). Descriptive statistics showed an increase in median accuracy from 35.00% in Round 1 to 60.00% in Round 4. Post-hoc analyses with Bonferroni adjustment revealed that accuracy in Round 1 was significantly lower than in Round 3 (MedDiff =  − 25.00%, 95% CI: − 45.00 to − 10.00%, Z =  − 2.771, adjusted *P* = 0.034) and Round 4 (MedDiff =  − 25.00%, 95% CI: − 40.00 to − 10.00%, Z =  − 2.771, adjusted *P* = 0.034). Although accuracy in Round 1 was also lower than in Round 2 (MedDiff =  − 20.00%, 95% CI: − 35.00 to − 9.88%, Z =  − 2.425, raw P = 0.015), this difference was not statistically significant after Bonferroni correction (adjusted *P* = 0.092). Moreover, no significant differences were observed among Rounds 2, 3, and 4 (all adjusted *P* > 0.05. The results were also shown in Fig. 3.

**Fig. 4:**
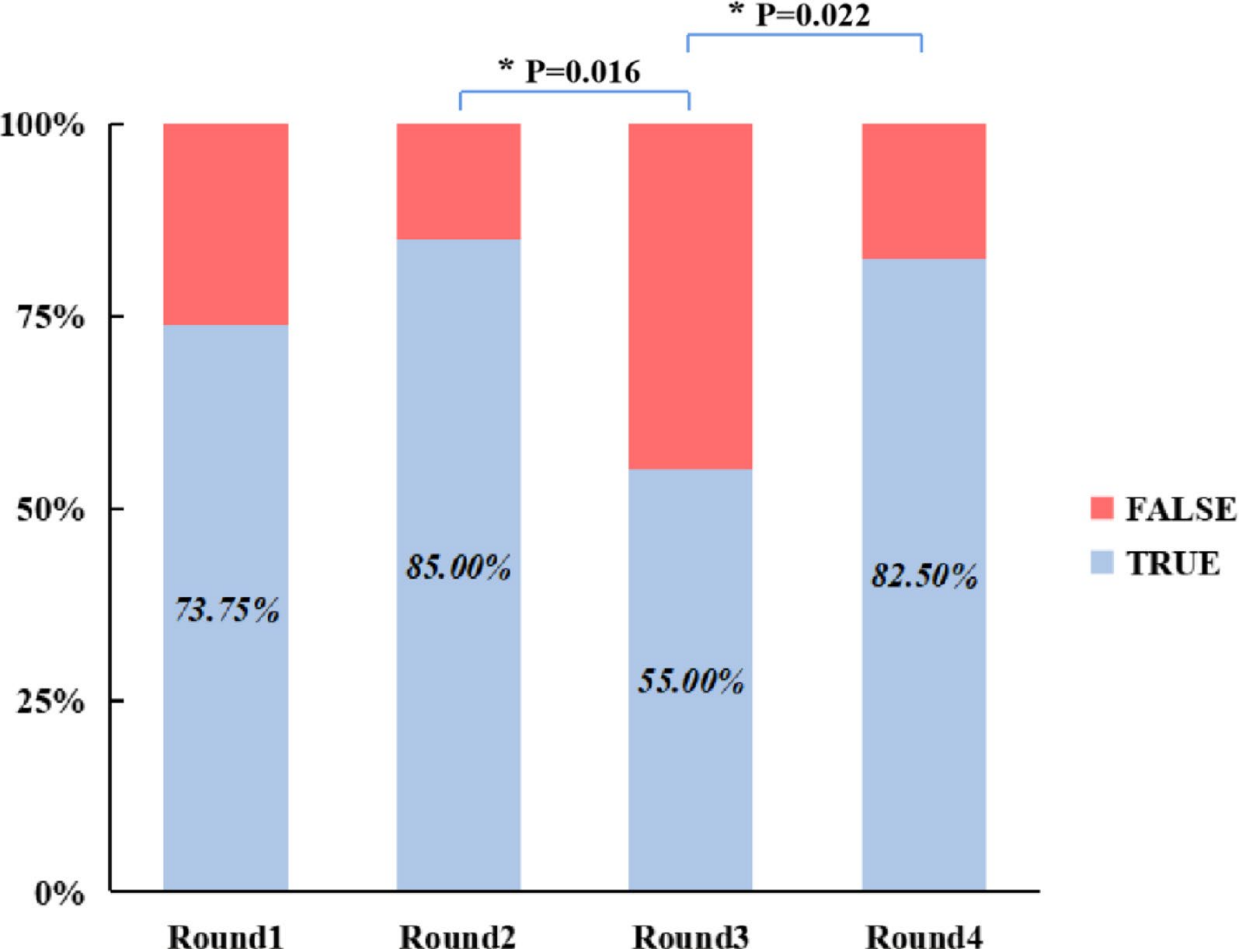
Comparison of classification accuracy across test rounds within the senior group. Statistically significant differences identified through post-hoc pairwise comparisons are indicated with brackets. Groups without brackets did not differ significantly. An asterisk (*) denotes that the *P*-value has been adjusted using the Bonferroni correction

The senior group exhibited distinct performance patterns. Accuracy rates from Round 1 to Round 4 were 73.75% ± 10.61% (median = 75.00%, IQR: 62.50–80.00%), 85.00% ± 10.69% (median = 90.00%, IQR: 72.50–90.00%), 55.00% ± 24.49% (median = 60.00%, IQR: 27.50–70.00%), and 82.50% ± 10.35% (median = 80.00%, IQR: 72.50–90.00%), respectively. Due to a violation of homogeneity of variances (P = 0.048), the Friedman test was similarly applied. A significant overall effect of testing rounds was found (χ^2^ = 14.162, *P* = 0.003). Subsequent pairwise comparisons showed that accuracy in Round 3 was significantly lower than in Round 2 (MedDiff =  − 30.00%, 95% CI: − 55.00 to − 10.00%, Z = 3.002, adjusted *P* = 0.016) and Round 4 (MedDiff =  − 20.00%, 95% CI: − 60.00 to − 5.00%, Z =  − 2.905, adjusted *P* = 0.022). The comparison between Rounds 2 and 4 revealed comparable classification accuracy (MedDiff = 10.00%, 95% CI: − 10.00 to 15.00%, Z = 0.097, adjusted *P* = 1.000). These findings collectively indicate that while senior residents performed equally well when independently using either conventional imaging or 3D models, their accuracy significantly declined when both modalities were used in combination. No other pairwise differences were statistically significant (all adjusted *P* > 0.05; Fig. 4).

#### Intra-round accuracy comparisons

**Fig. 5:**
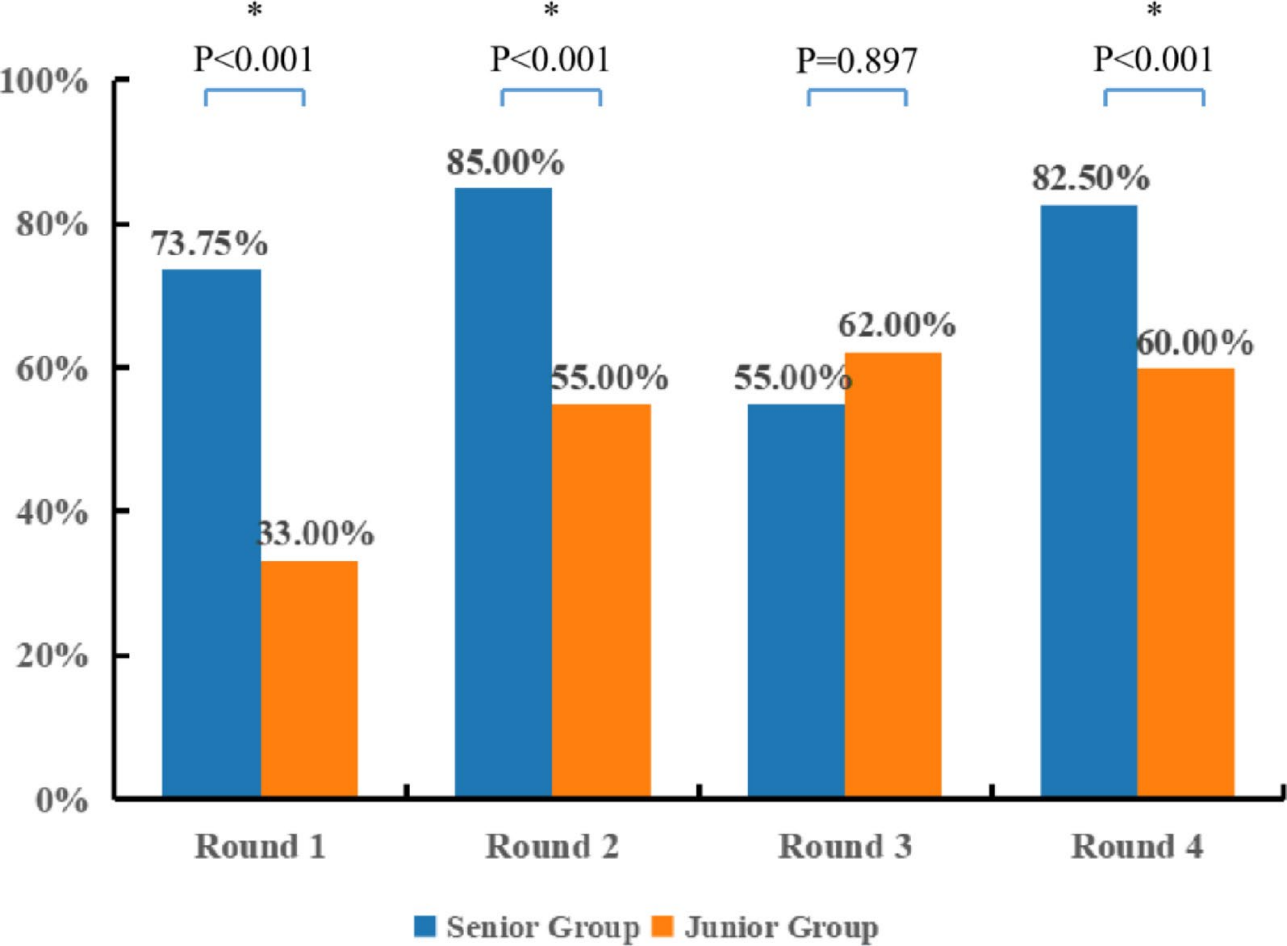
Comparison of classification accuracy between senior and junior groups in each round. Bars indicate the mean classification accuracy (%) for each group. Independent samples t-tests were used for comparisons in rounds 1, 2, and 4. For round 3, the Mann–Whitney U test was applied due to violation of the normality assumption in the junior group. An asterisk (*) denotes statistical significance at *P* < 0.05

Intra-round comparisons revealed that the senior group demonstrated significantly higher accuracy than the junior group in Rounds 1, 2, and 4 (73.75% ± 10.61% *vs* 33.00% ± 20.58%, 85.00% ± 10.69% *vs* 55.00% ± 11.79%, 82.50% ± 10.35% *vs* 60.00% ± 10.54%; *t* =  − 5.068, − 5.587, − 4.536, respectively; all *P* < 0.001). The corresponding effect sizes were substantial, with MD of 40.75% (95%CI: 23.71 to 57.80%), 30.00% (95%CI: 18.62 to 41.38%), and 22.50% (95%CI: 11.98 to 33.02%), respectively. In contrast, no significant difference was observed between senior (60.00%, IQR: 27.50–70.00%) and junior group (60.00%, IQR: 47.50–67.50%) in Round 3 (MedDiff = 0, U = 38.00, *P* = 0.897), indicating a notable convergence in performance between the two groups during this specific round. Detailed statistics are presented in Fig. 5.

### Time consumption

The median completion time for each round were as follows: Round 1, 385.00 s (IQR: 351.75–455.50); Round 2, 494.50 s (IQR: 401.75–611.00); Round 3, 1080.00 s (IQR: 704.25–1767.25); and Round 4, 705.50 s (IQR: 620.25–815.25). The Friedman test revealed a statistically significant difference in time consumption across the rounds (χ^2^ = 30.933, *P* < 0.001). Post-hoc pairwise comparisons with Bonferroni adjustment showed that time consumption in Round 1 was significantly shorter than in Round 3 (MedDiff = –695.00 s, 95% CI: − 1231.51 to − 387.00, Z =  − 5.164, adjusted *P* < 0.001) and Round 4 (MedDiff =  − 320.50 s, 95% CI: − 421.50 to − 232.00], Z =  − 3.615, adjusted *P* < 0.001). Similarly, Round 2 was significantly shorter than both Round 3 (MedDiff =  − 585.50 s, 95% CI: − 1150.01 to − 283.50, Z =  − 3.615, adjusted *P* < 0.001) and Round 4 (MedDiff =  − 211.00 s, 95% CI: − 344.50 to − 82.00, Z =  − 2.066, adjusted *P* = 0.039). These results indicate that 3D models, whether used alongside digital imaging data or independently, resulted in longer time consumption.

### Subjective feedback questionnaire results

**Fig. 6:**
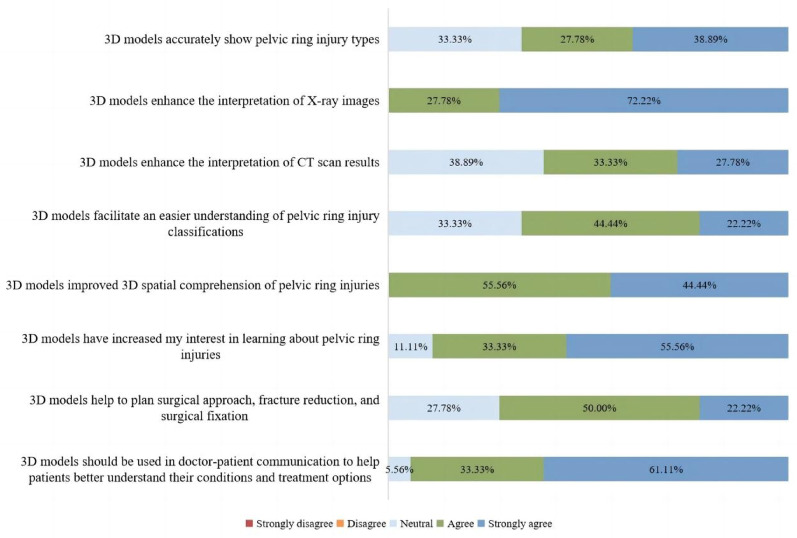
Overall results of participants’ subjective feedback on the utility of 3D printed models

None of the participants selected “disagree” or “strongly disagree” for any of the eight items, indicating their generally favorable views toward the 3D models. Notably, in terms of “enhancing X-ray interpretation” (Question 2) and “improving spatial understanding” (Question 5), all participants selected either “agree” or “strongly agree”. Similarly, strong positive responses were recorded for “increasing learning interest” (Question 6) and “facilitating doctor-patient communication” (Question 8), with approval rates exceeding 80%. However, lower levels of agreement were observed for “improving CT interpretation” (Question 3, 61.11%) as well as “accurately showing injury types” (Question 1, 66.67%) and “enhancing classification understanding” (Question 4, 66.67%). Interestingly, a more cautious attitude emerged regarding “preoperative planning assistance” (Question 7), where over one-quarter of participants (27.78%) selected a neutral response. A breakdown of the detailed results can be found in Fig. 6.

## Discussion

Numerous studies have demonstrated the efficacy of 3D models in medical education, particularly in enhancing spatial understanding and increasing participants’ interest [28, 31, 39–42]. As far as we know, the study by Yan et al. [36] remains the first and only investigation to specifically evaluate the utility of 3D printed models in pelvic fracture classification. Therefore, our study provides the second piece of evidence in this area and contributes to bridging the current research gap. Both inter-group and intra-group comparisons in this study revealed experience-stratified difference in the impact of 3D printed models on classification accuracy. Specifically, the models improved accuracy among junior residents but, paradoxically, led to decreased accuracy in the senior group. Another key finding was that 3D models, whether used independently or as adjuncts to digital imaging, significantly increased time expenditure. Furthermore, subjective feedback highlighted a strong preference for using 3D models as classification adjuncts rather than independent clinical decision-making tools. Their practical value in surgical planning has not gained unanimous support.

The fracture classification accuracy is a critical objective metric in assessing the clinical utility of 3D printed models. Current evidence demonstrates that 3D printed models can significantly improve orthopedic residents’ understanding of acetabular fractures and the accuracy of Letournel-Judet classification [34, 35]. Yan et al. [36] recently reported that 3D printed models can enhance classification accuracy among medical students in pelvic fractures. In our study, both the addition of 3D CT reconstructions (Round 2, 68.33%) and the use of 3D printed models alone (Round 4, 70.00%) significantly outperformed traditional 2D imaging alone (Round 1, 51.11%, Bonferroni-adjusted *P* < 0.05, Fig. 2). This finding aligns with the results of Lim et al. [34], who found that orthopedic residents using either CT or 3D printed models achieved better acetabular fracture classification accuracy than those relying solely on 2D images. Collectively, these results highlight the unique advantages of 3D visualization technologies, both digital reconstruction and physical models, in facilitating spatial understanding of complex anatomy. By providing intuitive spatial relationships, they effectively overcome the inherent limitations of 2D imaging in conveying depth and 3D perspective. This concept finds practical application in the recent work of Ramadanov et al. [43], who developed a reproducible technique for visualizing and delineating a patient-specific safe zone (Ramadanov-Zabler Safe Zone) for sacroiliac screw placement using CT-based 3D model and the corresponding 2D lateral projection. The feasibility demonstrated in their representative case strongly aligns with our findings, confirming that 3D models, even in digital form, are instrumental in analyzing intricate anatomical structures.

Surprisingly, our study revealed an experience-stratified difference of 3D printed models’ use. When all imaging modalities were combined, overall classification accuracy decreased rather than improved (Round 3: 58.89%, Fig. 2). Stratified analysis further revealed this trend was most pronounced in the senior group (Round 3 vs Round 2: MedDiff =  − 30.00%, 95% CI: − 55.00 to − 10.00%, adjusted *P* = 0.016; Round 3 vs Round 4: (MedDiff =  − 20.00%, 95% CI: − 60.00 to − 5.00%, adjusted *P* = 0.022; Fig. 4). In contrast, junior residents showed consistent improvement when 3D models were included, with classification accuracy increasing significantly from Round 1 to Round 3 (MedDiff =  − 25.00%, 95% CI: − 45.00 to − 10.00%, adjusted *P* = 0.034) and Round 4 (MedDiff =  − 25.00%, 95% CI: − 40.00 to − 10.00%, adjusted *P* = 0.034, Fig. 3), suggesting that junior residents can benefit more from the use of 3D printed models, whereas the multimodal information may have a negative effect on classification performance among senior residents. This result differs notably from that of Yan et al. [36], who reported that 3D printed models improved overall classification accuracy. One possible reason for this discrepancy is the difference in study populations: while our study involved orthopedic residents with varying levels of experience and a foundational understanding of pelvic fractures, Yan et al. [36] focused on fourth-year undergraduate medical students in a five-year clinical program, whose lower baseline knowledge may have allowed for greater improvement. However, from our perspective, this contrast actually reinforces our conclusion that the benefits of 3D printed models are more obvious among less experienced learners. Another contributing factor may be the different classification systems used in each study. Yan et al. [36] adopted the simpler, mechanism-based Young-Burgess system, while ours employed the more complex AO/OTA classification system with a greater number of types, groups, and subgroups, making our evaluation of 3D model efficacy more rigorous. Regarding the experience-stratified difference of 3D printed models’ efficiency, a possible explanation may be the different cognitive demand between junior and senior groups: for junior residents, 3D printed models help compensate for their limited spatial reasoning ability and thus improve their understanding of complex fracture patterns. Conversely, multimodal integration may cause redundancy and interference on senior residents, increasing cognitive load and decreasing accuracy.

Furthermore, the experience-stratified difference of 3D printed models in classification accuracy was also validated by our intra-round comparisons. In Rounds 1 and 2, where no 3D model was provided, senior group demonstrated significantly higher overall accuracy compared to the junior group (Fig. 5), highlighting the critical role of clinical experience. However, this experience advantage diminished notably with the addition of 3D printed models. In Round 3, which integrated all modalities, the overall accuracy between senior and junior groups converged (MedDiff = 0, *P* = 0.897), Fig. 5). These results further support our own conclusion of inter-round comparisons and also align with findings from Lim et al. [34], who used Fleiss’ Kappa test to assess interobserver agreement in acetabular fracture classification among residents with different training levels. Their study showed that the improvement in interobserver agreement after the application of 3D printed models was more pronounced in the junior group (from slight to fair agreement) than in the senior group (from fair to fair agreement), reinforcing the notion that 3D printed models offer clear educational value for junior residents, while potentially disrupting the established diagnostic strategies of senior residents, thus serving to narrow or even offset the experience gap. This “experience-compensating effect” may be attributed to the intuitive 3D visualization provided by 3D printed models, which helps less experienced individuals overcome their limitations in spatial reasoning. Conversely, for senior residents, the simplified anatomical representation of the 3D models may conflict with their well-established cognitive frameworks developed through years of interpreting 2D images. This expertise-interference phenomenon manifests as measurable accuracy reduction and therefore warrants special attention in clinical implementation. This finding has important implications for our clinical practice and education, as well as for future research: the use of 3D printed models should be precisely tailored to clinical practitioners’ level of experience in order to maximize their educational and diagnostic value, rather than applying them indiscriminately to all experienced groups.

Time consumption is another important metric for evaluating the utility of 3D printed models in fracture classification. In a study by Yan et al. [36], the group using 3D models consumed significantly less time to classify pelvic fractures (191.8 ± 37.8 s) compared to the group without 3D models’ assistance (238.6 ± 44.5 s; *P* < 0.0001), suggesting an improvement in decision-making efficiency. In contrast, our findings showed that both independent and adjunctive use of 3D printed models significantly increased the time consumption compared to conventional digital imaging alone. This discrepancy may primarily be attributed to differences in study populations. The study of Yan et al. [36] focused on medical students, most of which lacked strong radiographic interpretation skills, so the intuitive and tangible nature of 3D printed models may have enabled quicker recognition of fracture types and injury mechanisms. However, our participants were orthopedic residents with certain years of clinical experience and a foundational ability to interpret conventional imaging. They may experience cognitive interference when integrating tangible 3D models with established digital image interpretation proficiency, subsequently resulting in increased time consumption. However, this negative effect may diminish as they gain more experience with 3D models, warranting further investigation in future studies. Additionally, the different classification systems employed may also account for this discrepancy. Given the inherent complexity of the AO classification system for pelvic injuries, it is possible that 3D models indeed provide limited assistance in reducing decision-making time.

Learns’ feedback is a valuable approach for evaluating the educational significance of instructional tools [44, 45]. In our study, subjective feedback results showed a high overall level of acceptance toward 3D printed models among participants, particularly in enhancing spatial understanding and assisting with X-ray interpretation. All participants selected “agree” or “strongly agree” for these items (Fig. 6), consistent with findings from previous studies [46–48]. This indicates that the 3D nature of models effectively mitigates the challenges of translating 2D radiographs into spatial understanding, which is one of the major difficulties in medical education. Furthermore, over 80% of participants agreed or strongly agreed that 3D models were helpful in increasing learning interest and facilitating doctor-patient communication (Fig. 6), demonstrating the dual value of these models in both educational and communicative contexts [49, 50]. However, responses were more reserved regarding the models’ role in improving CT interpretation, accurately showing injury types, and enhancing classification understanding. This finding aligns with the observed decrease in accuracy among senior residents during Round 3 (imaging + 3D models), suggesting that 3D models may be of limited added value in these aspects for residents with well-developed skills in interpreting digital imaging. Additionally, the relatively low level of agreement concerning preoperative planning assistance suggests that participants currently view 3D models more as educational adjuncts rather than primary clinical decision-making tools.

Although this study yielded valuable findings regarding the role of 3D printed models in assisting AO/OTA classification of pelvic fractures, several limitations must be acknowledged. First of all, the limited sample size (18 participants who completed all testing rounds) restricts the statistical power and external validity of the results, particularly for subgroup comparisons. However, as a single-center study with standardized training and testing protocols, potential biases from inconsistent educational exposure were minimized. Future studies should expand to multi-center settings with a larger and more diverse participant pool to improve external validity. In addition, the study focused on a narrow range of pelvic fracture cases (10 in total), without covering all sub-types or incorporating other frequently used classification systems such as Young-Burgess or Tile systems. This may limit the generalizability of the findings and highlights the need for future research to include more fracture types and classification systems to better assess the robustness of 3D models across clinical scenarios. Moreover, the uniform 75% scale reduction of the models, while ensuring print compatibility, may impair visualization of linear fractures that could fall below the printer’s effective resolution. This resolution limitation particularly affects detection of subtle comminuted fragments and occult cracks in complex fracture patterns where such morphological details are crucial for accurate classification and clinical decision-making. Future studies should incorporate quantitative analysis of model fidelity and investigate its correlation with diagnostic performance to identify which anatomical features are most critical for educational and clinical utility. A further concern is the fixed sequence of testing rounds, which may have introduced a learning effect. While variations in imaging combinations were used to minimize this, a randomized crossover design in future investigations would provide a more rigorous evaluation of each modality’s independent impact. Finally, the subjective feedback was based on self-reported questionnaires, which may be influenced by expectation bias or prior exposure to 3D models, potentially overestimating their educational value, especially regarding cognitive engagement and motivation. To better combine subjective perceptions with objective outcome measures in future studies would offer a more comprehensive and reliable assessment of educational effectiveness. Overall, these limitations point to important areas for refinement in future research. Addressing them will not only enhance our understanding of the utility of 3D printed models but also guide their optimal integration into orthopedic training and clinical practice.

## Conclusions

In light of the acknowledged limitations above, including a fixed testing sequence, a small cohort, and an unbalanced case spectrum, this study offers preliminary insights into the utility of 3D printed models for pelvic injury AO/OTA classification. Our results identified experience-stratified difference in the effectiveness of 3D printed models on pelvic injury AO/OTA classification accuracy. Specifically, while the models enhanced performance among junior residents, they unexpectedly led to reduced accuracy of the senior group. Additionally, compared to using digital imaging alone, both independent and adjunctive use of 3D printed models was associated with increased time consumption, suggesting limited efficiency benefits in decision-making workflows. Moreover, subjective feedback indicated a strong preference for using 3D models as classification adjuncts rather than independent tools for clinical decision-making, with no clear consensus on their practical utility in surgical planning.

Taken together, while this study supports the potential value of 3D printed models in improving pelvic fracture classification, particularly for less experienced residents, it also highlights the importance of careful integration into clinical workflows and training programs. Further research is warranted to clarify their role and optimize their application across different levels of expertise and clinical contexts.

## Data Availability

All data in this study are available from the corresponding author on reasonable request.

## Abbreviations

3D: Three-dimensional
2D: Two-dimensional
CT: Computed tomography
DICOM: Digital imaging and communications in medicine
MD: Mean difference
MedDiff: Median difference
STL: Stereolithography
IQR: Interquartile range
SD: Standard deviation

## References

[1] Baker G, Diamond O. Pelvic fractures. Surg Infect (Larchmt). 2021;39(6):341–9. 10.1016/j.mpsur.2021.04.003.

[2] Küper MA, Trulson A, Stuby FM, et al. Pelvic ring fractures in the elderly. EFORT Open Rev. 2019;4(6):313–20. 10.1302/2058-5241.4.180062. 31312519 10.1302/2058-5241.4.180062 PMC6598730

[3] Incagnoli P, Puidupin A, Ausset S, et al. Early management of severe pelvic injury (first 24 hours). Anaesth Crit Care Pain Med. 2019;38(2):199–207. 10.1016/j.accpm.2018.12.003. 30579941 10.1016/j.accpm.2018.12.003

[4] Tullington JE, Blecker N. Pelvic Trauma. 2023 Aug 8. In: StatPearls [Internet]. Treasure Island (FL): StatPearls Publishing; 2024

[5] Baker JE, Werner NL, Burlew CC. Management of pelvic trauma. Surg Clin North Am. 2024;104(2):367–84. 10.1016/j.suc.2023.10.001. 38453308 10.1016/j.suc.2023.10.001

[6] Elamin MH, Elkaramany I, Salman LA, et al. The epidemiology of pelvic ring fractures in Qatar. Int Orthop. 2024;48(4):1097–103. 10.1007/s00264-024-06103-w. 38296877 10.1007/s00264-024-06103-w PMC10933172

[7] Davis DD, Foris LA, Kane SM, et al. Pelvic Fracture. 2023 May 1. In: StatPearls [Internet]. Treasure Island (FL): StatPearls Publishing; 2024

[8] Ramadanov N, Voss M, Hable R, Prill R, Salzmann M, Becker R. Comparative evaluation and ranking of anterior surgical approaches for acetabular fractures: a systematic review and network meta-analysis. Injury. 2025;56(4):112241. 10.1016/j.injury.2025.112241. 40154238 10.1016/j.injury.2025.112241

[9] Mennen AHM, Blokland AS, Maas M, et al. Imaging of pelvic ring fractures in older adults and its clinical implications-a systematic review. Osteoporos Int. 2023;34(9):1549–59. 10.1007/s00198-023-06812-9. 37286662 10.1007/s00198-023-06812-9 PMC10427539

[10] Yoo SJ, Hussein N, Peel B, et al. 3D modeling and printing in congenital heart surgery: entering the stage of maturation. Front Pediatr. 2021;9:621672. 10.3389/fped.2021.621672. 33614554 10.3389/fped.2021.621672 PMC7892770

[11] Huang J, Wang H, Yang Y, et al. 3D printing of foetal vascular rings: feasibility and applicability. BMC Pregnancy Childbirth. 2023;23(1):355. 10.1186/s12884-023-05683-6. 37194003 10.1186/s12884-023-05683-6 PMC10186654

[12] Si J, Zhang C, Tian M, et al. Intraoral condylectomy with 3D-printed cutting guide versus with surgical navigation: an accuracy and effectiveness comparison. J Clin Med. 2023;12(11):3816. 10.3390/jcm12113816. 37298011 10.3390/jcm12113816 PMC10253499

[13] Ma J, Chen Z, Liu S, et al. The application of 3D-printed oral stents in intensity-modulated radiotherapy for oropharyngeal cancer and their dosimetric effect on organs at risk. Eur J Med Res. 2023;28(1):367. 10.1186/s40001-023-01333-x. 37736754 10.1186/s40001-023-01333-x PMC10515031

[14] Tahmawy YA, Mohamed FS, Elfeki S, et al. Microbiological evaluation of conjunctival anopthalmic flora after using digital 3D-printed ocular prosthesis compared to conventional one: a randomized clinical trial. BMC Oral Health. 2023;23(1):1012. 10.1186/s12903-023-03746-w. 38110937 10.1186/s12903-023-03746-w PMC10729395

[15] Masada KM, Cristino DM, Dear KA, et al. 3-D printed fracture models improve resident performance and clinical outcomes in operative fracture management. J Surg Educ. 2023;80(7):1020–7. 10.1016/j.jsurg.2023.04.004. 37198080 10.1016/j.jsurg.2023.04.004

[16] Liang H, Zhang H, Chen B, et al. 3D printing technology combined with personalized plates for complex distal intra-articular fractures of the trimalleolar ankle. Sci Rep. 2023;13(1):22667. 10.1038/s41598-023-49515-1. 38114629 10.1038/s41598-023-49515-1 PMC10730506

[17] van der Lelij TJN, Marang-van de Mheen PJ, Kaptein BL, et al. Continued stabilization of a cementless 3D-printed total knee arthroplasty: five-year results of a randomized controlled trial using radiostereometric analysis. J Bone Joint Surg Am. 2023;105(21):1686–94. 10.2106/JBJS.23.00221. 37651549 10.2106/JBJS.23.00221 PMC10609712

[18] Grinčuk A, Petryla G, Masionis P, et al. Short-term results and complications of the operative treatment of the distal radius fracture AO2R3 C type, planned by using 3D-printed models: prospective randomized control study. J Orthop Surg (Hong Kong). 2023;31(2):10225536231195128. 10.1177/10225536231195127. 37620284 10.1177/10225536231195127

[19] Liu F, Lei Q, Cai L, et al. Efficacy comparison between iliosacral screw fixation of the posterior pelvic ring fracture with the assistance of modified percutaneous three-dimensional printing guide template and conventional fluoroscopy(in Chinese). Zhong Nan Da Xue Xue Bao Yi Xue Ban. 2023;48(11):1703–10. 10.11817/j.issn.1672-7347.2023.230122. 38432861 10.11817/j.issn.1672-7347.2023.230122 PMC10929951

[20] Eyiis E, Mathijssen NMC, Kok P, et al. Three-dimensional printed customized versus conventional plaster brace for trapeziometacarpal osteoarthritis: a randomized controlled crossover trial. J Hand Surg (European Volume). 2023;48(5):412–8. 10.1177/17531934221146864. 10.1177/17531934221146864 36650951

[21] Aiba H, Spazzoli B, Tsukamoto S, et al. Current concepts in the resection of bone tumors using a patient-specific three-dimensional printed cutting guide. Curr Oncol. 2023;30(4):3859–70. 10.3390/curroncol30040292. 37185405 10.3390/curroncol30040292 PMC10136997

[22] Müller DA, Stutz Y, Vlachopoulos L, et al. The accuracy of three-dimensional planned bone tumor resection using patient-specific instrument. Cancer Manag Res. 2020;12:6533–40. 10.2147/CMAR.S228038. 32801891 10.2147/CMAR.S228038 PMC7397560

[23] Gouin F, Paul L, Odri GA, et al. Computer-assisted planning and patient-specific instruments for bone tumor resection within the pelvis: a series of 11 patients. Sarcoma. 2014;2014:842709. 10.1155/2014/842709. 25100921 10.1155/2014/842709 PMC4101950

[24] Biscaccianti V, Fragnaud H, Hascoët JY, et al. Digital chain for pelvic tumor resection with 3D-printed surgical cutting guides. Front Bioeng Biotechnol. 2022;10:991676. 10.3389/fbioe.2022.991676. 36159695 10.3389/fbioe.2022.991676 PMC9493251

[25] Evrard R, Schubert T, Paul L, et al. Quality of resection margin with patient specific instrument for bone tumor resection. J Bone Oncol. 2022;34:100434. 10.1016/j.jbo.2022.100434. 35601663 10.1016/j.jbo.2022.100434 PMC9115318

[26] Park JW, Kang HG. Application of 3-dimensional printing implants for bone tumors. Clin Exp Pediatr. 2022;65(10):476–82. 10.3345/cep.2021.01326. 34942688 10.3345/cep.2021.01326 PMC9561186

[27] Tarca A, Woo N, Bain S, et al. 3D printed cardiac models as an adjunct to traditional teaching of anatomy in congenital heart disease-a randomised controlled study. Heart Lung Circ. 2023;32(12):1443–50. 10.1016/j.hlc.2023.09.021. 38007317 10.1016/j.hlc.2023.09.021

[28] Neijhoft J, Sterz J, Rüsseler M, et al. Evaluation of a 3D-printed hands-on radius fracture model during teaching courses. Eur J Trauma Emerg Surg. 2024;50(1):49–57. 10.1007/s00068-023-02327-4. 37524864 10.1007/s00068-023-02327-4 PMC10923998

[29] Kiesel M, Beyers I, Kalisz A, et al. A 3D printed model of the female pelvis for practical education of gynecological pelvic examination. 3D Print Med. 2022;8(1):13. 10.1186/s41205-022-00139-7. 35511353 10.1186/s41205-022-00139-7 PMC9069962

[30] Asghar A, Naaz S, Patra A, et al. Effectiveness of 3D-printed models prepared from radiological data for anatomy education: a meta-analysis and trial sequential analysis of 22 randomized, controlled, crossover trials. J Educ Health Promot. 2022;11:353. 10.4103/jehp.jehp_199_22. 36567994 10.4103/jehp.jehp_199_22 PMC9768753

[31] Potarazu D, Herur-Raman A, Cho EY, et al. A visuospatial and kinesthetic 3D-printed model of inguinal anatomy improves applied anatomy knowledge. Global Surg Educ. 2023;2(1):104. 10.1007/s44186-023-00184-8.

[32] Brumpt E, Bertin E, Tatu L, et al. 3D printing as a pedagogical tool for teaching normal human anatomy: a systematic review. BMC Med Educ. 2023;23(1):783. 10.1186/s12909-023-04744-w. 37864193 10.1186/s12909-023-04744-w PMC10589929

[33] Wu AM, Wang K, Wang JS, et al. The addition of 3D printed models to enhance the teaching and learning of bone spatial anatomy and fractures for undergraduate students: a randomized controlled study. Ann Transl Med. 2018;6(20):403. 10.21037/atm.2018.09.59. 30498730 10.21037/atm.2018.09.59 PMC6230865

[34] Lim PK, Stephenson GS, Keown TW, et al. Use of 3D printed models in resident education for the classification of acetabulum fractures. J Surg Educ. 2018;75(6):1679–84. 10.1016/j.jsurg.2018.04.019. 29929817 10.1016/j.jsurg.2018.04.019 PMC6346736

[35] Goyal S, Chua C, Chen YS, et al. Utility of 3D printed models as adjunct in acetabular fracture teaching for orthopaedic trainees. BMC Med Educ. 2022;22(1):595. 10.1186/s12909-022-03621-2. 35918716 10.1186/s12909-022-03621-2 PMC9344721

[36] Yan M, Huang J, Ding M, et al. Three-dimensional printing model enhances correct identification and understanding of pelvic fracture in medical students. J Surg Educ. 2023;80(3):331–7. 10.1016/j.jsurg.2022.10.016. 36470716 10.1016/j.jsurg.2022.10.016

[37] Meinberg EG, Agel J, Roberts CS, et al. Fracture and dislocation classification compendium-2018. J Orthop Trauma. 2018;32(Suppl 1):S1–170. 10.1097/BOT.0000000000001063. 29256945 10.1097/BOT.0000000000001063

[38] Landis JR, Koch GG. The measurement of observer agreement for categorical data. Biometrics. 1977;33(1):159–74. 10.2307/2529310. 843571

[39] Nicot R, Druelle C, Chazard E, et al. Three-dimensional printing model enhances craniofacial trauma teaching by improving morphologic and biomechanical understanding: a randomized controlled study. Plast Reconstr Surg. 2022;149(3):475e–84e. 10.1097/PRS.0000000000008869. 35196687 10.1097/PRS.0000000000008869

[40] Karsenty C, Guitarte A, Dulac Y, et al. The usefulness of 3D printed heart models for medical student education in congenital heart disease. BMC Med Educ. 2021;21(1):480. 10.1186/s12909-021-02917-z. 34496844 10.1186/s12909-021-02917-z PMC8424617

[41] Wu C, Luo M, Liu Y, et al. Application of a 3D-printed eye model for teaching direct ophthalmoscopy to undergraduates. Graefes Arch Clin Exp Ophthalmol. 2022;260(7):2361–8. 10.1007/s00417-021-05538-w. 35038015 10.1007/s00417-021-05538-w

[42] Lane JC, Black JS. Modeling medical education: the impact of three-dimensional printed models on medical student education in plastic surgery. J Craniofac Surg. 2020;31(4):1018–21. 10.1097/SCS.0000000000006567. 32433138 10.1097/SCS.0000000000006567

[43] Ramadanov N, Zabler S. Ramadanov-Zabler safe zone for sacroiliac screw placement: a CT-based computational pilot study. J Clin Med. 2025;14(10):3567. 10.3390/jcm14103567. 40429562 10.3390/jcm14103567 PMC12112452

[44] Adarkwah MA. The power of assessment feedback in teaching and learning: a narrative review and synthesis of the literature. SN Soc Sci. 2021;1(3):75. 10.1007/s43545-021-00086-w.

[45] Röhl S, Bijlsma H, Schwichow M. Can feedback from students to teachers improve different dimensions of teaching quality in primary and secondary education? A hierarchical meta-analysis. Educ Assess Eval Acc. 2025;37:35–71. 10.1007/s11092-024-09450-9.

[46] Ben Awadh A, Clark J, Clowry G, et al. Multimodal three-dimensional visualization enhances novice learner interpretation of basic cross-sectional anatomy. Anat Sci Educ. 2022;15(1):127–42. 10.1002/ase.2045. 33369254 10.1002/ase.2045

[47] Zhang Y, Wang H, Yi J, et al. A novel 3D printed model for educating medical students on limb fractures: a randomized controlled preliminary study. J Orthop Surg Res. 2024;19(1):624. 10.1186/s13018-024-05088-x. 39367473 10.1186/s13018-024-05088-x PMC11451155

[48] Agarwal A, D Souza A, Jyostna B, et al. Introducing 3D printed models of fractures in osteology learning improves clinical reasoning skills among first-year medical students: a pilot study. BMC Med Educ. 2025;25(1):190. 10.1186/s12909-025-06746-2. 39915742 10.1186/s12909-025-06746-2 PMC11800631

[49] Kahsai EA, O’Connor B, Khoo KJ, et al. Improving patient understanding of femoroacetabular impingement syndrome with three-dimensional models. J Am Acad Orthop Surg Glob Res Rev. 2024;8(5):e24.00116. 10.5435/JAAOSGlobal-D-24-00116. 38722846 10.5435/JAAOSGlobal-D-24-00116 PMC11081616

[50] Kargul M, Skórka P, Gutowski P, et al. Empowering EVAR: revolutionizing patient understanding and qualification with 3D printing. J Cardiovasc Dev Dis. 2024;11(11):365. 10.3390/jcdd11110365. 39590208 10.3390/jcdd11110365 PMC11594954

